# Experimental Evaluation of a 3D-Printed Fluidic System for a Directional Anemometer

**DOI:** 10.3390/s20154094

**Published:** 2020-07-23

**Authors:** Andrea Ria, Alessandro Catania, Paolo Bruschi, Massimo Piotto

**Affiliations:** 1 Department of Ingegneria dell’Informazione, University of Pisa, 56122 Pisa, Italy; andrea.ria@ing.unipi.it (A.R.); alessandro.catania@ing.unipi.it (A.C.); paolo.bruschi@unipi.it (P.B.); 2 Institute of Electronics, Computer and Telecommunication Engineering, National Research Council of Italy, 56122 Pisa, Italy

**Keywords:** 3D printing, differential pressure anemometer, directional wind sensor, sensor miniaturization, low-power sensors, 2D anemometer

## Abstract

An evolution of a previously proposed anemometer capable of detecting both the magnitude and the direction of the wind on a plane is proposed. The device is based on a recently formalized principle, consisting of combining the differential pressures measured across distinct diameters of a cylinder to estimate the wind velocity and incidence angle. Differently from previous sensors based on the same principle, the proposed anemometers use 3D printing to fabricate the channel structure that calculates the pressure combination in the fluidic domain. Furthermore, commercial sensors with low power consumption are used to read the two pressures that result from the fluidic processing. The whole fabrication procedure requires inexpensive equipment and can be adopted by small enterprises or research laboratories. Two original channel structures, predicted by previous theoretical work but never experimentally validated, are proposed. The results of detailed experiments performed in a wind tunnel are reported.

## 1. Introduction

Anemometers are devices designed to detect the velocity vectors of gas streams. Traditional fields of application for anemometers are weather forecasting, assistance in navigation and airport operations. Recently, the importance of detecting the magnitude and, possibly, the direction of air streams has emerged in several innovative fields. Precision agriculture [1] requires the monitoring of several parameters, among which the wind velocity is recognized to be an important factor [2,3]. Even the annual production of honey was found to be affected by the wind intensity [4]. In the field of robotics, anemometers are being proposed as fundamental sensors to be combined with gas detectors in order to enable autonomous vehicles to locate gas sources [5,6,7]. UAVs (unmanned aerial vehicles) represent another emerging field that is stimulating the development of a new generation of anemometers. UAVs can be used, for example, to produce 3D maps of the wind distribution for studies of atmosphere phenomena [8,9] and to predict the evolution of forest fires [10]. Wind is also a primary source of disturbance on UAV flight parameters: the prediction of wind effects has been proposed as a way to improve trajectory and attitude control [11]. Although wind intensity and direction can be inferred by the knowledge of the UAV attitude and other flight parameters (indirect approach) [10,12], direct measurement of the wind velocity by an onboard anemometer may help to improve the robustness of the wind estimate and is hindered only by the limitations of the currently available sensors [13]. 

These innovative applications introduce new specifications for the anemometers. In most of the mentioned applications, the anemometer should be able to detect both the magnitude and direction of the wind velocity vector, at least on a plane. Often, especially for gas source tracking applications, it is required to detect small wind velocities, even below 1 m/s. Furthermore, miniaturization and low power consumption are also of primary importance for onboard use by small battery-powered vehicles or wireless sensor network (WSN) nodes. Another crucial requisite is the cost, especially when the anemometer has to be integrated into relatively inexpensive platforms. 

Different approaches are being proposed to fabricate small anemometers with directional capabilities. Use in fast-moving vehicles (e.g., UAVs) rules out mechanical devices, such as cup and vane anemometers, whose moving parts would be too sensitive to inertial forces. The category of wind sensors that is being used most frequently in the mentioned studies is that of ultrasonic anemometers. These are based on the time-of-flight principle and can be designed to detect two components of the wind velocity vector (2D anemometers) or even all three components (3D anemometers). Among their strengths are the absence of moving parts and environmental robustness. The use of ultrasonic transducers based on MEMS (micro-electro mechanical systems) allows for a good degree of miniaturization and low power consumption [14]. On the other hand, their size cannot be reduced below several centimeters, since sufficient spacing between the transducers should be maintained to reduce the impact of parasitic effects such as unwanted reflection and shadowing caused by the transducer bodies and by their supporting elements. An alternative solution is represented by acoustic resonance sensors, based on the phase shift caused by the wind on an acoustic standing wave generated in an open chamber. These sensors, invented and commercialized by FT Technologies [15], are practically absent from the scientific literature, although they are beginning to be placed onboard UAVs for their small size and robustness [9].

From the point of view of miniaturization, the wind sensors that show the best potential are thermal anemometers, which largely benefit from the advent of MEMS technologies. Initially developed to detect small flow rates inside pipes with extremely low power consumption, MEMS thermal anemometers are being proposed to directly detect the wind intensity and direction [16]. A major weakness of devices in this class is represented by the small dimensions of the thermal elements (heaters and temperature probes), resulting in reduced mechanical robustness and high sensitivity to even single dust particles or water droplets. This is an intrinsic weakness [17] since the reduction of the sensor elements to the size of several microns is necessary for the mentioned reduction of the power consumption. Furthermore, dedicated micromachining technologies are often required [16,17,18,19,20,21], resulting in high fabrication costs unless very large production volumes can be envisioned. The use of simpler technologies with off-the-shelf components is feasible but results in larger power consumption [22] and response times on the order of seconds in contrast with the millisecond time-constants of MEMS sensors. The use of plastic or ceramic substrates with low thermal conductivity, combined with metal deposition and etching steps, has been proposed to partially simplify the fabrication flow while keeping dimensions small to mitigate power consumption [23,24].

Another very popular approach is the DP (differential pressure) method, consisting of detecting the pressure differences induced by the wind stream when it hits a body of proper shape. This is the principle exploited by the well-known Pitot tubes, where the pressure difference is measured between the tip of a tube-shaped body and a region of undisturbed air. This method is not well suited to measuring small wind velocities, due to the necessity of detecting very small differential pressures. Recently, the development of compact, low-cost MEMS differential pressure sensors allowed the extension of the DP approach also to wind velocities below 1 m/s. In 2009, we proposed a method to detect the magnitude and direction of the wind on a plane (2D anemometer) by combining the pressure differences developed by the wind across several pairs of points placed on the outer surface of a cylinder [25]. The advantage with respect to previous DP approaches that combined different pressure differences developed across bluff bodies [26,27] is that a linear combination was performed in the fluidic domain, leading to the requirement of only two differential pressure sensors. For the first prototype developed in [25], and following devices based on the same principle [28,29,30], very small pressure differences were acquired by monitoring the air flow rate induced by the pressure difference inside a capillary duct. This operation was accomplished using non-commercial MEMS flow sensors previously developed by our group [31]. While in [25,28,29,30], the position of the point pairs at which the pressure was sampled and the weights that characterize the linear combination of differential pressures were determined on the basis of a numerical optimization procedure, a theoretical explanation of the approach was given only in 2018 [32], using spectral analysis. Different approaches that use several differential pressure samples measured across a cylinder but infer the wind direction and velocity from analytical approximations of the pressure around the stagnation point have been proposed [8,33]. The use of a sphere in place of the cylinder was proposed in [34].

In this work, we present a further development of the work done around the DP approach formalized in [32]. The differences with respect to the previous practical implementations of the approach [25,28,29,30] are the following: (i) use of 3D printing technology for the fabrication of the fluidic components that operate the linear combination of pressures; (ii) use of commercial differential pressure sensors in order to facilitate the development of low-cost products; (iii) experimentation of different angle/weight combinations than in previous works. The use of 3D printing has been already proposed in the field of anemometry to fabricate Pitot tubes [35], Cobra probes [36], strain-based anemometers [37,38], hotwire flow sensors [39] and a capacitive pressure-based anemometer [40]. 

In this work, 3D printing (stereolithography) is used to fabricate complex fluidic structures and for the large diffusion of this technique that should favor reproducing the results presented here by other research groups.

## 2. Sensor Description

### 2.1. Principles of Operation

**Figure 1:**
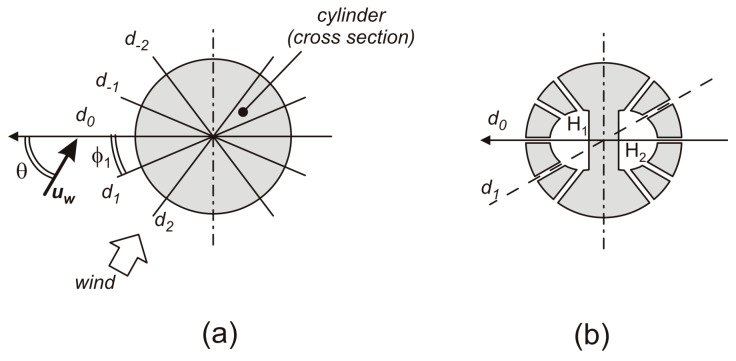
(**a**) Cylinder cross-section showing the elements which are useful in understanding the operating principle; (**b**) Channel configuration used to perform linear combination of the diametric pressures in the fluid-dynamic domain.

Figure 1a depicts the cross-section of a cylinder, taken by cutting the cylinder with a plane perpendicular to the cylinder axis. In the rest of this paper, we will use the term “diameter” to indicate a straight-line segment that passes through the center of the circle and whose endpoints lie on the circumference. For the sake of simplicity, depending on the context, we will use the word “diameter” to mean also the width of the circle, equal to twice the radius. In Figure 1a, a reference axis is shown; the diameter that lies along the reference axis will be indicated with *d_0_* (reference diameter). The cylinder is exposed to an air stream (wind) whose velocity vector **u_w_** is parallel to the plane of the cross-section and forms an angle θ with the reference axis. An even number of additional diameters are considered together with the reference diameter in such a way that diameter *d*_k_ forms an angle ϕ_k_ with the reference axis. The configuration is symmetric with respect to *d*_0_, so that for each diameter *d*_k_, we have also a symmetrical diameter *d*-_k_ at angle –ϕ_k_.

A given diameter configuration is identified by the number of diameters and by the angles that each diameter forms with the reference diameter (*d*_0_). The number of diameters is expressed by an integer *N*, such that the diameter set is *d*_−N_, ... , *d*_−1_, *d*_0_, *d*_1_, ... , *d*_N_, forming angles –ϕ_N_, .....−ϕ_1_, 0, ϕ_1_, ...., ϕ_N_. Then, the total number of diameters is 2*N* + 1, with a total of *N* independent angles. Let us define the diametric pressure as the pressure difference developed by the wind across a given diameter. A diametric pressure is represented by the symbol *P_D_*(ϕ,θ,*u_w_*), where ϕ is the angle formed by the diameter with *d_0_*, while θ and *u_w_* are the wind direction (see Figure 1a) and magnitude. Following [32], it is possible to associate a set of *N*+1 weights *w_0_*,..*w_N_* to a given diameter configuration, such as that of Figure 1a, and calculate the following linear combination of the diametric pressures:

(1) $$P_{C}\left(\theta ,u_{w}\right)=\sum_{k=-N}^{N}w_{k}P_{D}\left(\phi_{k},\theta ,u_{W}\right)\;\;\;\;\;\;$$

where *w_−k_* = *w_k_*. In [32], it was shown that, with a proper choice of angles ϕ_k_ and weights *w_k_*, it is possible to obtain a *P_C_* function whose dependence on the wind direction (θ) is a good approximation of a cosine function. In this way, we can write the following:

(2) $$P_{C}\left(\theta ,u_{w}\right)≅H\left(u_{w}\right)\;\cos\left(\theta \right)\;\;\;\;\;$$

where *H*(*u_w_*) is a monotonic function of the wind magnitude *u_w_* only. A 2D anemometer can be simply obtained by using two diameter arrangements like the one in Figure 1a, mounted in such a way that their reference diameters (*d*_0_) are orthogonal. Consider a reference system with the *x*-axis parallel to the central diameter of one arrangement (the “X section”) and the *y*-axis parallel to diameter *d*_0_ of the other arrangement (the “Y section”). Then, applying (1) to both sections, we can obtain two combined pressures *P_X_* and *P_Y_*, respectively, that show the following dependence on *u_w_* and θ:

(3) $$\left\{ \begin{array}{l} P_{X}\left(\theta ,u_{w}\right)≅H\left(u_{w}\right)\;\cos\left(\theta \right)\\ P_{Y}\left(\theta ,u_{w}\right)≅H\left(u_{w}\right)\;\sin\left(\theta \right)\;\; \end{array}\right.\;\;$$

where θ is the angle formed by the wind direction with the *x*-axis. Clearly, θ and *u_w_* can be easily found from *P*_X_ and *P*_Y_ by the following:

(4) $$\left\{ \begin{array}{l} \theta =\arctan\left(P_{X},P_{Y}\right)\\ H\left(u_{w}\right)=\sqrt{P_{X}^{2}+P_{Y}^{2}}\;\; \end{array}\right.\;\;$$

Due to the monotonic behavior of *H*(*u_w_*), it is possible to calculate *u_w_* using a calibration table or a proper fitting function. In [32], a theoretical explanation of Equation (2) is given on the basis of spectral analysis. Different sets of angles and weights that provide optimal approximation of the cosine behavior are reported in [32]. Arrangements with a larger number of diameters (i.e., larger *N*) generally give a better approximation and a larger interval of wind velocities where the approximation holds. Two different approaches are proposed: uniform weights (UW), characterized by *w*_o_ = *w*_1_ = .... *w_N_*, and uniform diameter spacing (UDS), where the diameters are evenly spaced across the 0–90° interval while the weights are not uniform. In the case of UW, it is necessary to find the optimal angles that, for a given number *N*, provide an optimum cosine approximation. In the case of UDS, the angles are fixed, and optimization is obtained by a proper set of weights. More details are given in [32]. 

A straightforward implementation of Equation (1) is placing one pressure sensor at the end of each diameter of the set. Diametric pressures, obtained by simple differences of two couples of pressure readings, can be combined by simple algebraic calculations. In this way, a large number of pressure sensors is required. For example, in the case of only three diameters per section (*N* = 1), the total number of sensors is 12. This number can be halved by using differential pressure sensors to acquire the diametric pressures directly, but the sensor count is still relatively high, especially for complex diameter arrangements (*N*>1). An important simplification was proposed in [25,28,29,30], where combination Equation (1) was performed in the fluidic domain, using a structure similar to that depicted in Figure 1b. An exhaustive explanation of the mechanism is given in [32]. Briefly, two cavities, H_1_ and H_2_ in Figure 1b, are connected to the cylinder’s outer surface by means of channels of small diameter. The points where the channels end at the outer surface are the extremes of the diameters chosen to implement the approach described above. For the sake of clarity, this correspondence is shown only for diameter d_1_ in Figure 1b. Considering laminar flow inside the channels, it can be shown that the pressure inside the cavities is a weighted average of the pressure values picked up at the points where the channels end on the outer surface [32]. Finally, if we take the pressure difference between chambers H_1_ and H_2_, we obtain a quantity equal to *P_C_*(θ,*u_w_*) in Equation (1) with the following:

(5) $$\sum_{k=-N}^{N}w_{k}=1\;\;\;\;\;\;$$

By this approach, only two differential pressure sensors are required, greatly simplifying the device, reducing size and cost, and simplifying calibration. Different weights can be obtained by using channels of different diameter or length. We have chosen to leave the channel diameter constant and vary the length, since this option requires a coarser fabrication resolution.

### 2.2. Geometry of the Fluid-Dynamic Elements (Heads)

In all previous experimental works, the channel structure was fabricated using a computer-controlled milling machine. The drawback of this subtractive manufacturing approach is that complex internal cavities and channel configurations could only be obtained by assembling separate cylinder sections together. Even with this solution, limitations still apply when full 3D structures should be fabricated. Furthermore, alignment between the X and Y sections of the anemometer and leakage containment are critical operations. 

**Figure 2:**
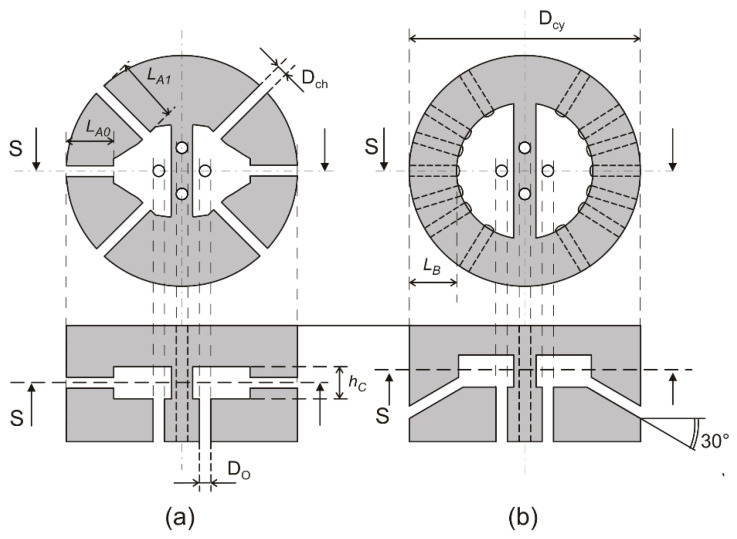
Transverse (top) and longitudinal (bottom) cross-sections of the designed channel/cavity combinations: (**a**) shows the UDS, *N* = 1 configuration, while (**b**) shows one of the two UW, *N* = 3 structures used in the experiments. The main dimensions are indicated.

Here, we have used a 3D printer to fabricate in a single piece both the X and Y sections of the cylinder. The object obtained in this way has been called the “head” of the cylinder. We have chosen to test two configurations that were proposed in [32] but have not yet been used in experimental works. The transverse and longitudinal sections of the structures are shown in Figure 2. Note that only the lower section is shown in the figure (X section), while the upper section of the head is identical but rotated by 90° along the cylinder axis. 

The first structure, shown in Figure 2a, implements a UDS configuration with *N* = 1 (three diameters). Different channel lengths have been obtained by the particular shape of the cavities visible in the figure. The angle between the central diameter (*d*_0_) and the lateral diameters (*d*_1_ and *d*_−1_) is 45° in this structure. The proper weight ratio *w*_1_/*w*_0_ is cos(45°), obtained by setting the *L_A0_ /L_A1_* ratio equal to cos(45°). The second structure, shown in Figure 2b, is a UW configuration with *N* = 3 (seven diameters). The requirement of uniform weights translates into equal length for all channels. As a result, the cavities are more regular than in the previous structure. Another difference is that the channels in the UW structure have an inclination of 30° with respect to the cylinder transverse plane. We made this choice to reduce the probability of water droplets reaching the internal cavities through the channels. We have designed two different UW structures, differing only in the length of the channels (2 mm and 4 mm). The structure in Figure 2b is the 4 mm structure. In the 2 mm structure, the cavities are proportionally larger, due to the shorter channels. The angles formed by the diameters (*d*_1_, *d*_2_, *d*_3_) with the central diameter are reported in Table 1, where data from structures presented in previous works are also included for comparison. Channel inclination was not applied to the UDS configuration (Figure 2a) because, due to the different length, the channels would have crossed the outer surface at different distances from the cylinder top. This, combined with the finite length of the cylinder, would have introduced possible additional causes of non-ideality. 

The cylinder diameter (*D*_cy_ in Figure 2) is set to 20 mm for all types of structures. Vertical channels, visible in the longitudinal sections of Figure 2 and represented as circular holes in the transverse sections, are used to connect the cavities to the pressure sensors, placed on the bottom of the cylinder. The upper section (Y section), placed over the structures shown in Figure 2, is connected to its individual pressure sensor through channels (indicated with dashed lines) that pass through the thick membrane that separates the two cavities of the lower section, avoiding any interaction with the X section. As a result, two pairs of holes are present on the bottom face of the heads. These hole pairs will be indicated as X and Y ports, respectively, in the remainder of this paper.

Other dimensions indicated in Figure 2 are reported in Table 2 for the three structures examined in this paper. The H7L and H7S structures are different only in the lengths of the channels that connect the internal cavities to the outer surface. Note that the actual channel length for these structures is greater than *L_B_* due to the 30° inclination shown in Figure 2b, since L_B_ is the length of the channel projection on the transverse cross-section. The three heads that appear in Table 2 are composed by the stacking of two orthogonal channel/cavity structures with the corresponding geometry indicated in the table. 

### 2.3. Fabrication of the Fluid-Dynamic Elements (Heads) 

The fabrication of the three heads has been performed by means of stereolithography, using the 3D printer “FORM 2” of Formlabs [41]. The material used was the “Resin Clear version 04” (methacrylate-based photopolymer) provided by Formlabs. The vertical resolution (single-layer thickness) of the printing process was 50 μm, while the minimum feature size on the X-Y plane was 150 μm. The total fabrication time was around 4 h for all heads. After laser polymerization, the heads were rinsed in isopropanol for 20 min to remove excess resin. This step, performed by the form-wash tool provided by Formlabs, was particularly important to prevent channel clogging from excess resin. The size of the resin bath allowed the fabrication of up to four heads in parallel. 

**Figure 3:**
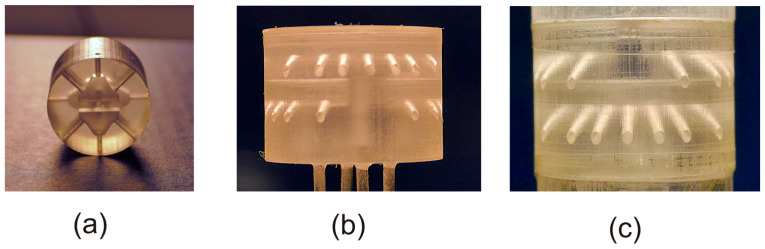
Photographs of heads with different channel configurations. (**a**) H3L head; (**b**) H7S head with stainless steel needles fitted to the pressure ports; (**c**) H7L head, mounted in the cylinder structure. Different points of view have been used for the various heads to show the non-regular shape of the H3L head and the channel inclination of the H7S and H7L heads.

Design of the heads was carried out using the open source program FreeCAD [42], producing output files in STL (STereoLithography) format, ready to be imported into the proprietary printer management software. Figure 3 shows photographs of the three different heads used in the experiments described in this paper. 

### 2.4. Architecture of the Prototypes

**Figure 4:**
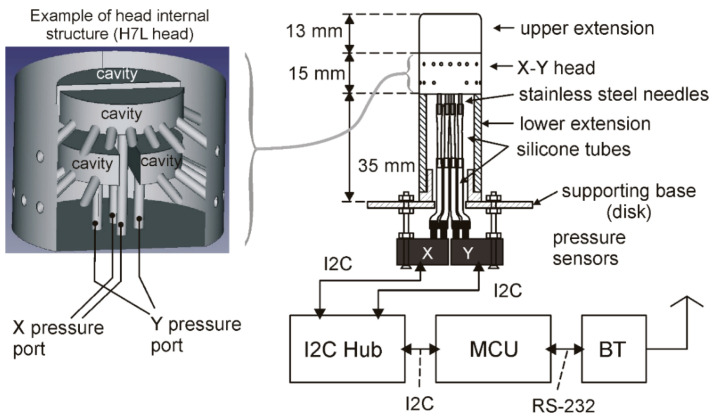
Representation of the device structure. On the left, a perspective view of the internal structure of a H7L head is shown. On the right, the whole structure of the sample is represented. The main dimensions of the cylinder structure are indicated.

Figure 4 illustrates the structure of the devices used in the experiments described in this paper. On the left, the internal structure of the heads is shown through a perspective view directly extracted from the FreeCAD design. The particular head shown in the figure is the H7L head. The X and Y sections and the pressure ports that allow access to the internal cavities of the individual sections are clearly visible. The heads are combined with other, simpler pieces to form the cylinder structure, as shown on the right. In particular, a top cylindrical piece, indicated with “upper extension” in the figure, has been placed on top of the heads to prolong the cylinder structure, reducing the effect of discontinuities at the top border of the heads. To further reduce discontinuity effects, the upper extension ended with a chamfer. A hollow cylinder, indicated with “lower extension” in Figure 4, was connected to the bottom face of the heads to continue the cylinder structure and cover the connection to the four pressure ports. Stacking of the lower extension, head, and upper extension forms a continuous cylinder structure. The upper and lower extensions were secured to the head by using adhesive tape in order to facilitate replacement of the heads. Connection to the pressure ports was accomplished by inserting stainless steel needles into the holes in the bottom faces of the heads, as visible in Figure 3b. The needles were secured to the heads by means of cyanoacrylate glue, carefully deposited around the needle’s outer surface before insertion into the pressure ports and around the junction perimeter. 

Connection to the pressure sensors was accomplished by means of silicone tubes. Two different diameters were required to fit the head pressure ports to the input ports of the pressure sensors, as schematically shown in the figure. The lower extension was split into two parts in order to simplify the manual connection of the head pressure ports to the silicone pipes.

The cylinder was joined to a circular plate providing support to the pressure sensors. Differential pressure sensor model SDP 810 by Sensirion [43] was used to detect *P_X_* end *P_Y_* (see Equation (3)). The full-scale pressure of sensors SDP 810 is ±500 Pa, while the resolution is around 0.015 Pa (calculated considering the 16-bit output data resolution). Each SDP 810 sensor requires around 12.5 mW from a 3.3 V power supply. Note that the estimated *P_X_* and *P_Y_* peak value for a wind velocity of 1 m/s is in the order of 1 Pa [32], thus pressure sensors with resolutions well below 1 Pa are required in order to enable operation at low wind velocities. 

The pressure sensors are read with a general purpose interface based on the MSP430i2041 microcontroller (Texas Instruments, Dallas, TX, USA). In particular, an I2C hub was required to independently communicate with the two sensors, which could not be connected to the same I2C bus since they are shipped with the same I2C address. The I2C hub was implemented as a distinct printed circuit board (PCB) module, based on the PCA9518 integrated circuit of Texas Instruments. Connection from the microcontroller unit (MCU) to a personal computer was implemented using the RN42 Bluetooth module by Microchip. The three components of the electronic interface shown in Figure 4 were designed by the authors and built as three separate printed circuit boards (PCBs). 

**Figure 5:**
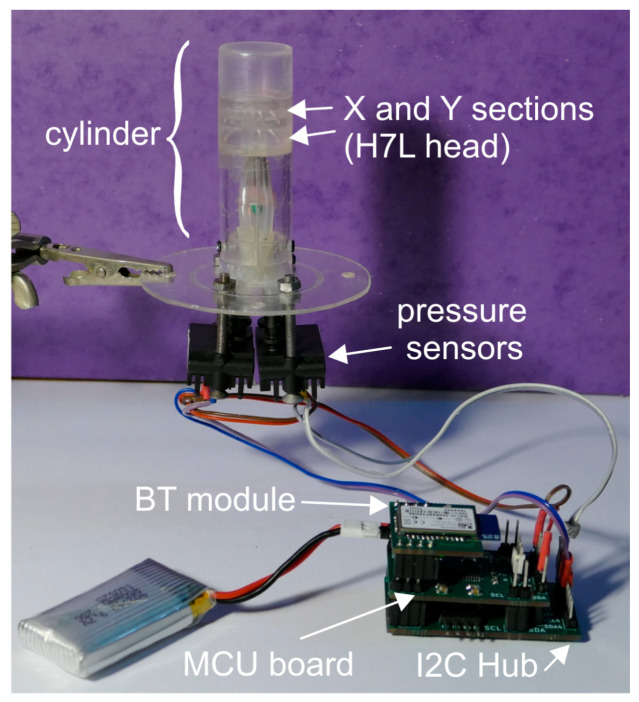
Photograph of the sensor with the wireless readout interface.

A photograph of the whole device is shown in Figure 5, where the various components represented in Figure 4 are clearly indicated. 

## 3. Results

### 3.1. Experimemental Setup and Data Processing

The measurements were performed using a small wind tunnel, consisting of a 1.2 m long circular pipe with 10 cm diameter. An electronically controlled fan allowed the air speed inside the tunnel to be set from 0.1 to around 8 m/s. Precise measurement of the air velocity was accomplished by means of a reference hot-wire anemometer (Model Testo AG 425, Testo SpA, Milan, Italy). Rotary components of the air flow due to the effect of the fan blades were reduced by filling a whole section of the pipe following the fan with an array of pipes of smaller diameter (2 cm), for a total length of 10 cm. The anemometers under test were inserted into the wind tunnel through a hole at nearly 20 cm from the exhaust end of the tunnel. The anemometers were secured to a sample holder that allowed complete 360-degree rotation around the cylinder axis, with a resolution of 1 degree. If not otherwise specified, the cylinder axis was perpendicular to the wind tunnel axis (i.e., to the wind velocity). Inclination tests were performed by introducing a tilt angle with respect to the perpendicular condition. A maximum inclination angle of 30 degrees was allowed by the sample-holder. In all the experiments, the sensitive sections of the cylinder were placed in the center of the wind tunnel, where the velocity is the highest. The reference hot-wire anemometer was placed as closely as possible to the height of the cylinder active sections. Displacements of ± 1 cm around the centerline did not cause significant differences in the responses of both the reference anemometer and devices under test. In the inclination tests, the height of the anemometer sample-holder was varied to keep the active section on the wind-tunnel centerline.

Pressure data acquisition was controlled by a program running on a personal computer that communicates with the anemometer through the Bluetooth link. For each measurement, a total of 16 acquisitions is averaged across a time span of nearly 2 s. By this procedure, the total noise (peak-to-peak) superimposed on the differential pressure measurements was found to be 0.02 Pa in a condition of zero wind velocity. Raw data were processed by means of scripts written with the Python language. The data were preliminarily corrected in order to take into account the pressure drop occurring along the connecting pipe. It is worth mentioning that, in conformity with other products having such a small detection limit, the SDP 810 sensors are actually thermal flow meters that infer the differential data from the mass flow rate through a capillary pipe. This is analogous to the approach that we proposed in [25,28,29,30], where we used highly miniaturized research-grade MEMS flow sensors [31]. The fact that a flow exists in the pipes that connect the head to the pressure sensors results in a significant difference between the actual differential pressure at the head ports and the sensor reading. A procedure to take into account this error is described in an application note available on the Sensirion website. Due to the irregular characteristics of the connecting pipes and the presence of bends, we preferred to directly calibrate the combination of SDP 810 sensors and connecting pipes. To this aim, we used an air flow line equipped with a precision mass flow controller to establish a constant differential pressure across two sections of a relatively large pipe (0.8 cm diameter), separated by a narrow obstruction. The differential pressure sensor to be calibrated (either the P_X_ or P_Y_ sensor) was connected across these two sections, through the actual pipe configuration that connects it to the pressure ports of the anemometer heads, including the stainless steel needles. The pressure across the same points is measured by means of a reference pressure sensor (another SPD 810 device) which, differently from the sensor to be calibrated, is connected by means of large and short pipes so that it can be considered free from pressure loss. Measurements performed at different pressures were collected into look-up tables, one for each channel, which were interpolated using spline functions. The offset of both sensors (of the order of 0.02 Pa) was acquired and subtracted from the measurements. The values of *P_X_* and *P_Y_*, acquired and corrected by this procedure, will be indicated with *P_XC_* and *P_YC_*, respectively, in the rest of this paper.

### 3.2. Results of Measurements

The three head configurations listed in Table 2 have been experimentally characterized and compared over a wind velocity range spanning from 0.9 to 8 m/s. The heads were combined with other components to form the device structure shown in Figure 4 and Figure 5. All additional parts were re-used, so that switching from one configuration to another was accomplished by simply changing the head. This operation was simplified by the use of adhesive tape for the connection of the parts that form the cylinder. For the sake of simplicity, detailed data resulting from the experiments are shown only for heads H3L and H7L. Data obtained with head H7S are shown in the final plot regarding measurement errors, which better summarizes the wind sensor performance and allows comparison between the different configurations.

**Figure 6:**
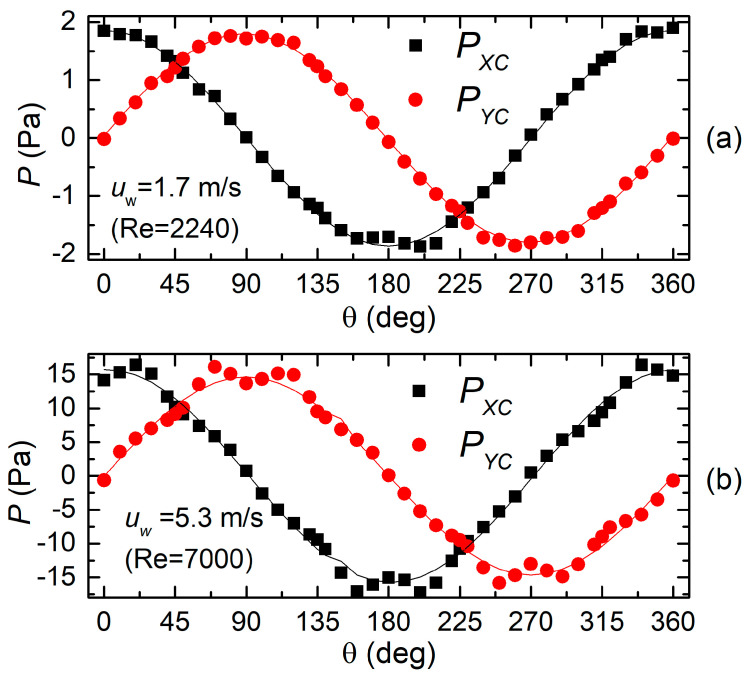
Pressure differences produced by the X (P_XC_) and Y sections (P_YC_) by the H3L head for 1.7 (**a**) and 5.3 m/s (**b**) wind velocity.

Figure 6a,b show the dependence of *P_XC_* and *P_YC_* on the wind direction θ (see Figure 1 for the meaning of angle θ) for the H3L head. The experimental data, represented by square and circular symbols, refer to two different wind velocities, namely 1.7 (Figure 6a) and 5.3 m/s (Figure 6b). The Reynolds numbers shown inside the figures have been calculated according to the formula:

(6) Re=ρairuwDcyμair

where *ρ_air_* and *μ_air_* are the air density and dynamic viscosity, respectively, while *D_cy_* is the cylinder diameter, equal to the head diameter given in Table 2. The Reynolds number is presented to facilitate comparison with the theoretical results described in [32]. Solid lines represent the best sinusoidal fits. Note that *P_XC_* and *P_YC_* follow a sinusoidal behavior with sufficient accuracy at the smaller velocity, while significant deviations are visible at the higher velocity setting. Deviations are larger around the peaks of the respective sinusoids.

Plots in Figure 6a,b is well representative of the behavior of the H3L head: approximation of the sinusoidal dependence is acceptable at low wind velocities, with errors that progressively grow as the velocity is increased. 

**Figure 7:**
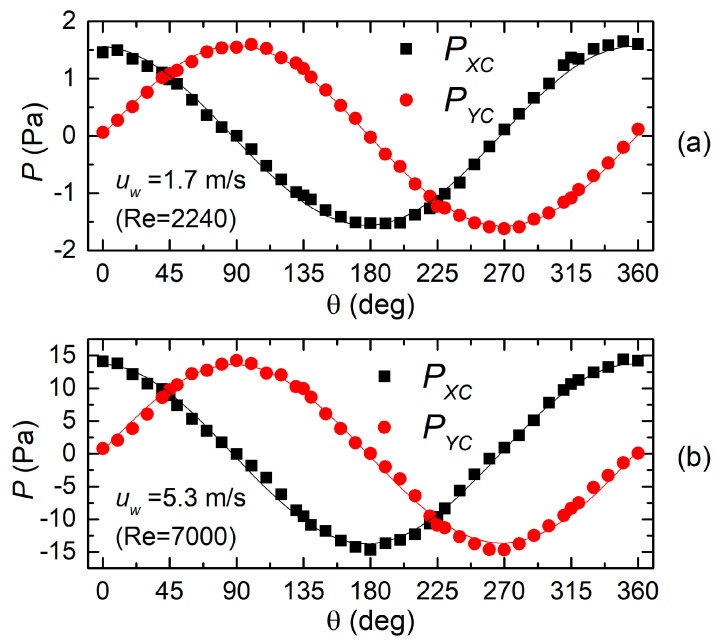
Pressure differences produced by the X (P_XC_) and Y sections (P_YC_) by the H7L head for 1.7 (**a**) and 5.3 m/s (**b**) wind velocity.

The same experiments performed with the H7L head produced the results shown in Figure 7a,b. The main difference that can be observed is the much better approximation of the sinusoidal behavior. Even at the higher velocity, deviation from the sinusoidal fits are modest. Note that the peak magnitude of the pressure is approximately the same for the two heads under test. The H7S head behaves much like the H7L configuration, with slightly larger deviations from the sinusoid.

**Figure 8:**
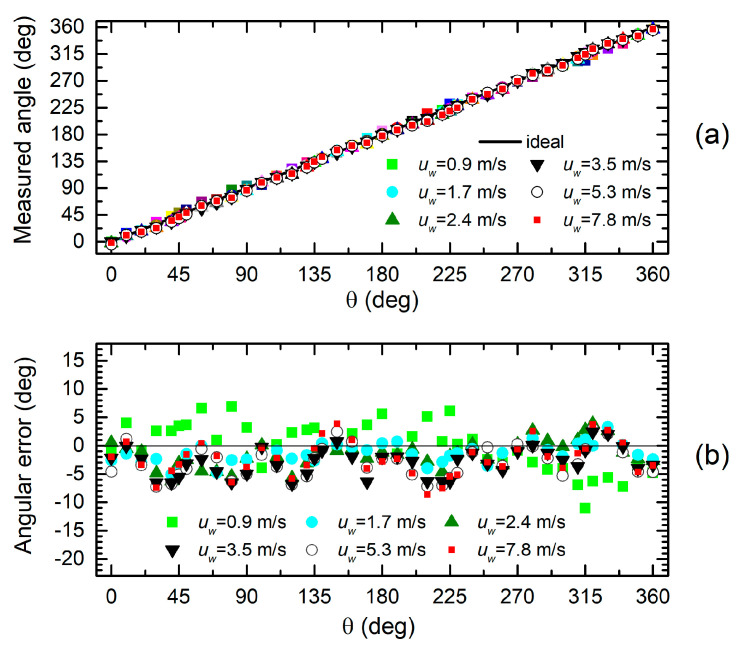
Measured angle (**a**) and angular error (**b**) as a function of the actual wind direction (θ) for the H3L head.

In order to understand how the observed discrepancies affect the sensor accuracy, we have used Equation (4) with *P_X_* = *P_XC_* and *P_Y_* = *P_YC_* to estimate the angle θ (measured angle) and the quantity *H*(*u_w_*), which we expect to be independent of θ. The capability of the H3L head to detect the wind direction is represented in Figure 8a,b, where the results of experiments performed over the whole explored wind range are shown. Figure 8a presents the measured angle as a function of the actual wind direction, read on the sample holder goniometer. A substantial agreement between the actual and measured quantity can be observed. The measurement errors are better highlighted by Figure 8b, which shows the difference between the actual and measured angle across the whole 360-degree range of wind directions. An oscillatory trend for the error can be observed, except for the 0.9 m/s data, which, due to the much smaller signal level, are likely to suffer from additional error from noise and other inaccuracies of the pressure sensors. The angular error varies from roughly −11 to +7 degrees. This uncertainty band reduces significantly if the 0.9 m/s data are excluded.

**Figure 9:**
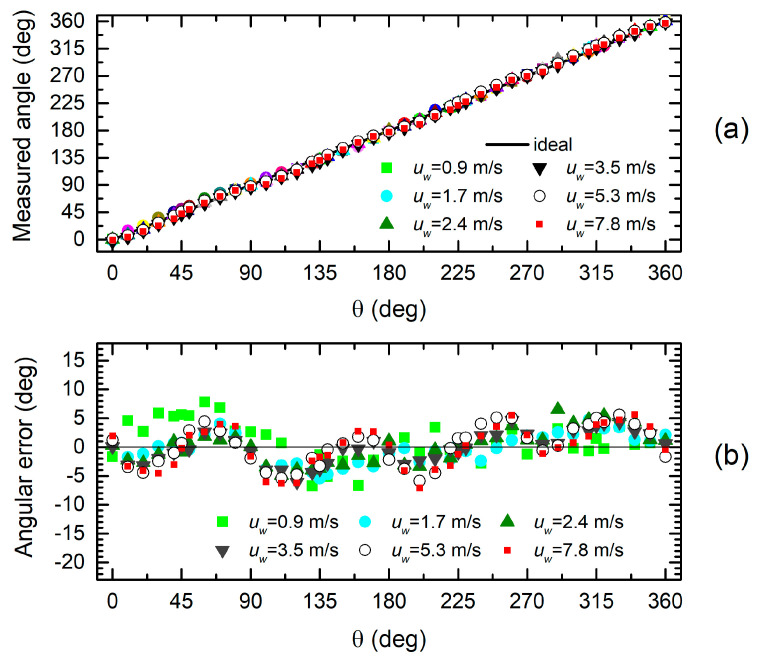
Measured angle (**a**) and angular error (**b**) as a function of the actual wind direction (θ) for the H7L head.

Angle estimation data for the H7L head are shown in Figure 9a,b. Comparison with the H3L head (Figure 8) does not show an important improvement in terms of angular accuracy, in spite of the much better fit of the sinusoidal behavior illustrated by Figure 6 and Figure 7. This can be explained by observing that the larger discrepancies visible in Figure 6 for the H3L heads are mostly located where the pressure value is close to the peak. With simple arguments of error propagation theory, we can find that the error on the estimated angle is given by the following:

(7) $$\Delta \theta =\frac{P_{X}\Delta P_{Y}-P_{Y}\Delta P_{X}}{P_{X}^{2}+P_{Y}^{2}}$$

where *P_X_* and *P_Y_* are the pressures that would be given by an ideal sinusoidal behavior (solid lines in Figure 6 and Figure 7), Δθ is the angular error, and Δ*P_Y_*, Δ*P_X_* are the pressure errors with respect to the ideal (sinusoidal) dependence. The consequence of Equation (7) is that the error on one of the two pressures is weighted by the value of the other pressure. As a result, errors located around the peaks give a negligible contribution to the angular error, since the other pressure is nearly zero. 

**Figure 10:**
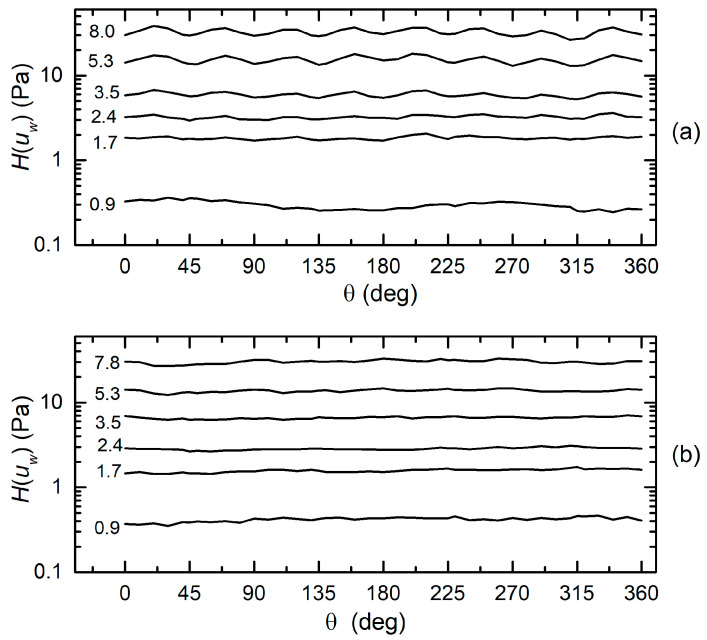
Estimated value of *H*(u_w_) as a function of the wind direction (θ) for the H3L head (**a**) and H7L head (**b**). Numbers close to the curves indicate the wind velocity *u*_w_ (m/s).

The situation is different when the estimate of quantity *H*(*u*_w_) is considered. In the case in which the sinusoidal laws represented by Equation (3) are strictly respected, the value of *H*(*u*_w_), estimated from *P_XC_* and *P_YC_* through Equation (4), would be independent of the wind direction θ. This would allow the use of *H*(*u*_w_) as an indication of the wind velocity magnitude, which could be estimated from *H*(*u*_w_) with simple expressions, as suggested in [32], or using an empirical calibration curve. The results of the estimation of *H*(*u*_w_) are shown in Figure 10a,b for the H3L and H7L head, respectively. 

The much larger dependence on the wind direction exhibited by the H3L head is clearly visible. Obviously, this is a consequence of the larger discrepancies exhibited by *P_XC_* and *P_YC_* for the H3L head. Differently from the case of the angle estimation, the expression of *H*(*u*_w_) is more sensitive to errors located in the proximity of peaks. 

**Figure 11:**
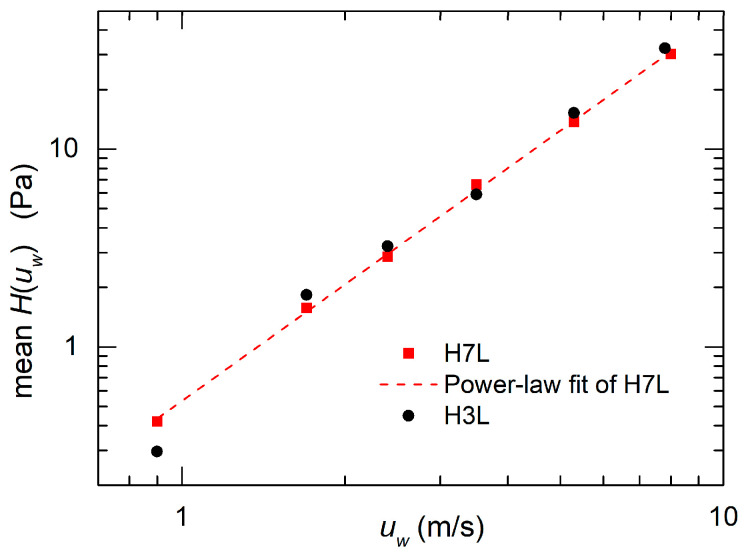
Mean value of *H*(u_w_) across the full 360° angle interval, as a function of the wind velocity for the H3L and H7L head. The good agreement with a power law fit (see the text) is apparent for the H7L head.

It is interesting to observe how the quantity *H*(*u_w_*) depends on the wind velocity magnitude. To eliminate the effect of the oscillations visible in Figure 10, we have averaged *H*(*u_w_*) across the full 360-degree angle range. The result is shown in Figure 11 for both the H3L and H7L head. A straight line fit for the H7L data is indicated. 

The excellent approximation of the fit indicates that *H*(*u_w_*) follows a power law dependence, as predicted in [32]. The fit has not been drawn for the H3L data since they do not appear to be aligned along a straight line. Considering the power law estimated from the fit of Figure 11, the expression that can be used to calculate the wind velocity from *H*(*u_w_*) is as follows:

(8) $$u_{w}=A\cdot H^{\beta }\;\;\;\;$$

where *A* = 1.38 and β = 0.511 with *u_w_* in m/s and *H* in Pa. 

**Figure 12:**
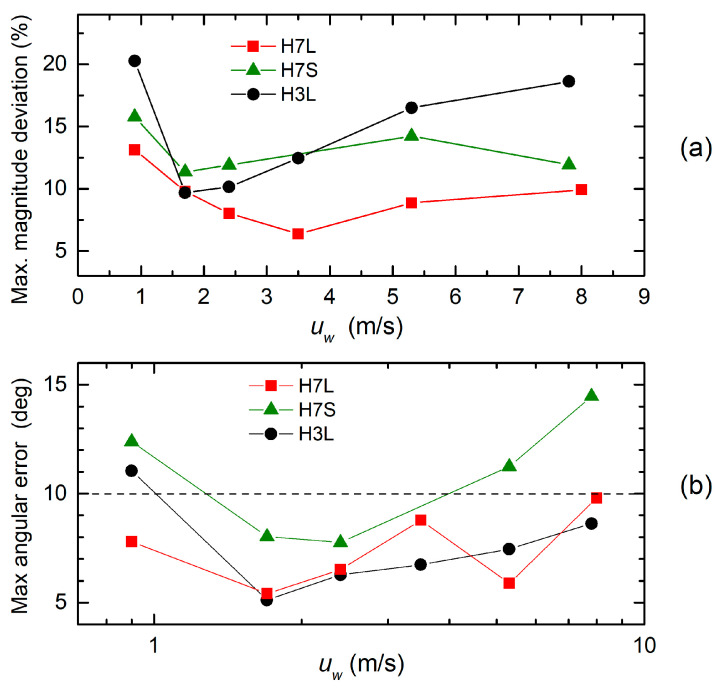
Magnitude (**a**) and angular (**b**) errors as a function of the wind velocity for all the heads under test. For both errors, the maximum value across the full 360° angular interval of wind directions is shown.

The accuracy that it is possible to obtain from the various heads tested in this paper is summarized in Figure 12, which shows the angular and magnitude error as a function of the magnitude of the wind velocity for heads H3L, H7L, and H7S. Each error point is calculated by taking the worst (largest) error measured by sweeping the wind direction across the full 360-degree range. As a measure of the magnitude error, we considered the maximum relative deviation of *H*(*u_w_*) from the average value, corresponding to the peak value of the oscillations visible in Figure 10.

**Figure 13:**
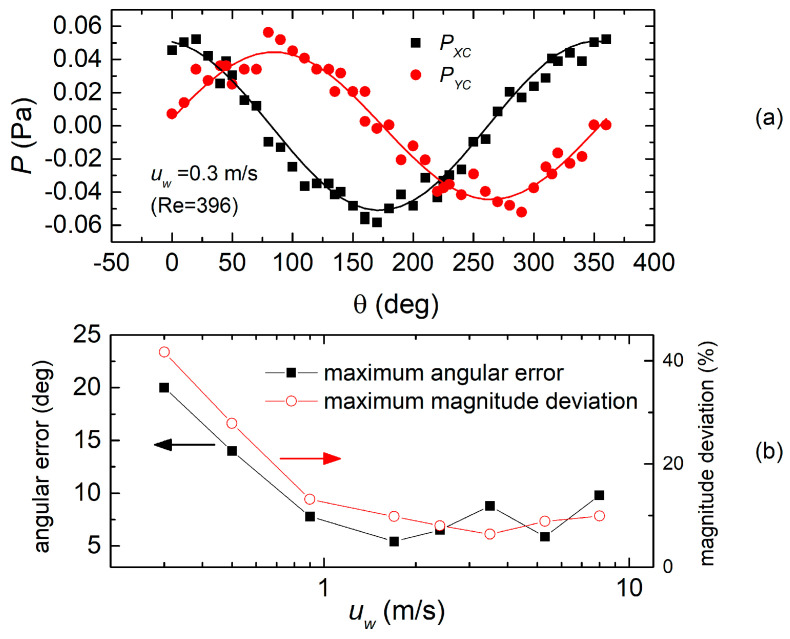
(**a**) Pressures *P_XC_* and *P_YC_* as a function of the wind direction for the H7L head and a wind velocity of 0.3 m/s. (**b**) Magnitude and angular errors as a function of the wind velocity for the H7L head, including measurements at 0.3 and 0.5 m/s.

For velocities smaller than those represented in Figure 11 and Figure 12, the pressure differences to be measured become so small that the errors from the sensors cannot be neglected. This is clearly proven by Figure 13a, where pressures *P_XC_* and *P_YC_* are shown as the function of the wind direction for the H7L head and a wind velocity of 0.3 m/s. 

The peak value of the pressure (0.05 Pa) at this velocity is less than three times the measured peak-to-peak noise (0.02 Pa). Figure 13b shows the angular and magnitude error plots for the H7L heads, where two points at 0.5 and 0.3 m/s have been added with respect to the same plots in Figure 12. The steep error increase for velocities smaller than 0.9 m/s is due to the dominance of the sensor noise. From Equation (7), it is possible to find the peak-to-peak value θ_n-pp_ of the noise superimposed on the angle estimation, considering that the equivalent noise pressures of sensors X and Y can be considered two independent random processes with same peak-to-peak value *P*_n-pp_:

(9) $$\theta_{n-pp}=\frac{P_{n-pp}}{H}$$

where *θ*_n-pp_ is in radians and *H* is given by Equation (4). At small wind velocities, *H* decreases according to a square law, while *P*_n-pp_ is clearly unaffected. Then, the fluctuation of the angle estimation grows larger and larger as *u_W_* is decreased and, eventually, becomes the dominant contribution to the error. The same consideration can be easily extended to the contribution of *P*_n-pp_ to the estimation of *H*. 

**Figure 14:**
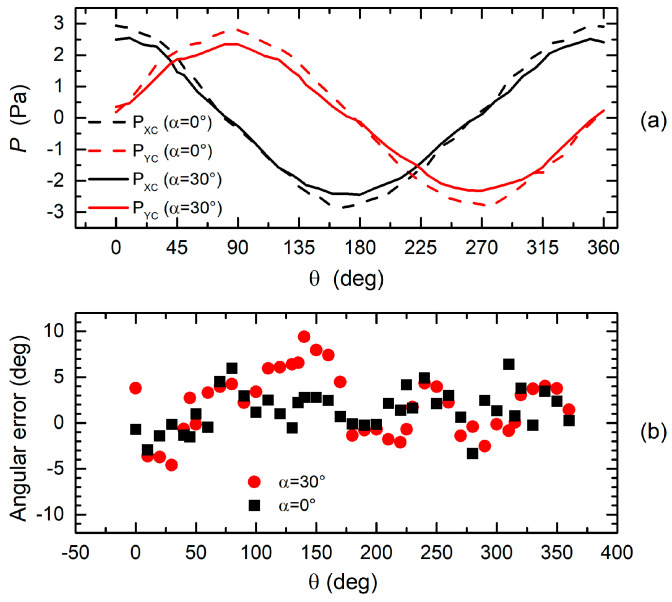
(**a**) Comparison between the measured *P_XC_* and *P_YC_* pressures for *u_W_*=2.4 m/s in the case of 0° and 30° angles formed by the cylinder axis and perpendicular to the plane of the wind velocity. (**b**) Angular error as a function of the wind direction for the inclination angles of 0° and 30°.

Finally, we have investigated the effect of the inclination of the cylinder axis with respect to the wind velocity vector. The results of experiments performed with the H7L head and a velocity of 2.4 m/s are shown in Figure 14. In particular, Figure 14a shows pressures *P_XC_* and *P_YC_* as a function of the wind direction in the case of a cylinder axis perpendicular to the plane of the wind velocity (α = 0) and for an inclination of 30 degrees (the tip of the cylinder was moved downstream). The attenuation of the pressure curves is close to cos(30°), which suggests that the situation is equivalent to considering only the velocity component on the plane perpendicular to the cylinder axis. Estimation of the wind direction performed with the inclined cylinder, shown in Figure 14b, is marked by only a slightly larger error than in the case of zero inclination. 

## 4. Discussion

The three heads tested in this work implement two different configurations formalized in [32]. In particular, the H3L head follows the UDS approach, with *N* = 1 (three diameters) per section, while the H7L and H7S represent different cases of the UW configuration with *N* = 3 (seven diameters). The difference between H7S and H7L is the length of the channels that connect the internal cavities to the cylinder outer surface. The errors produced by the proposed anemometers and summarized in Figure 12 are quite larger than the prediction that can be found in [32] for the same channel configurations and the same Reynolds number range. This result is in line with previous experimental tests [25,28,29,30], based on the channel configurations summarized in Table 1. The main reason, as already pointed out in [32], is the short length of the channels, which is in contrast with our hypothesis of a fully developed laminar flow. The possibility of calculating the linear combination of pressures represented by Equation (1) requires a linear dependence between the pressure drop across the channels and the mass flow rate through them. This is guaranteed only if the flow is laminar along the full length of the channels. Even if the Reynolds number of the flow inside the channels is small enough for a laminar flow to exist, a region of perturbed flow is present at the point where the channels end at the cylinder’s outer surface. The depth of this region can be several times the channel radius and grow longer at higher velocities. Comparing the H7L and H7S heads, which differ only in the channel length, it is clear that the longer channels of the H7L configuration produce smaller errors across the whole velocity range.

As far as the H3L head is concerned, deviations from the ideal behavior predicted in [32] for the same configuration are even larger and show up clearly in the poor approximation of the sinusoidal behavior visible in Figure 6. It should be observed that, in the UDS configurations, the approximation of the sinusoidal behavior relies on precise ratios between the weights, which, in the proposed implementation, would require a precise dependence between the fluidic conductance and the channel lengths. In turn, this needs a fully developed laminar flow inside the whole length of the channels. In addition, the H3L configuration is based on a smaller number of diameters with respect to heads H7L and H7S, and this contributes to increasing the error. Figure 12 confirms that the large deviations from the sinusoidal behavior have little impact on the angular accuracy, which is similar for the H3L and H7L heads, while it clearly impacts the independence of the *H*(*u_w_*) quantity from the angle (Figure 12a). In this respect, the H3L head performances are very similar to those of the H7S head, which has much shorter channels. The practical consequence is that the H3L and H7S heads will provide an estimate of the wind magnitude that depends more on the angle than the H7L head. The latter presents also the most regular dependence of the *H* magnitude on the wind magnitude, allowing simple determination of the latter.

It is useful to compare the anemometers presented in this paper with earlier devices based on the same principle, fabricated with a fully custom procedure. The maximum angular error and relative magnitude deviation are reported in Table 3 for the H7L and H3L heads and for three different channel configurations proposed in previous works (lines 3-5 of the table). A direct comparison between fabrication approaches is not possible, due to the different channel configurations. However, three considerations can be drawn: (i) UDS approaches, which require the implementation of different weights in the pressure combination and provide worse accuracies, independently of the order (*N*) and of the fabrication approach; (ii) the performances obtained with the H7L head are only slightly worse than those obtained with similar UW configurations and custom fabrication technology; (iii) in all cases, the errors are significantly larger than the prediction shown in [32].

The results obtained with the H7L heads indicate that combination of 3D printing and off-the-shelf pressure sensors is a viable approach to fabricating low-cost directional anemometers for applications that do not require high accuracies. On the other hand, the fact that, both in this work and in previous studies, a large discrepancy with respect to the theory presented in [32] was found indicates that further investigation is required to improve this class of devices. For example, longer channels could be designed, exploiting the potentiality of 3D printing, which here allowed the fabrication of inclined channels (H7L and H7S heads), which is not possible with the milling machine used in our previous works [25,28,29,30].

**Figure 15:**
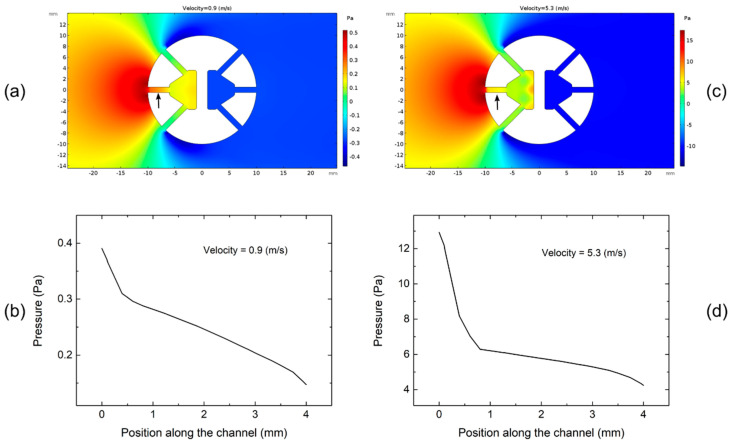
(**a**) Pressure distribution around the cylinder and inside the channels for wind velocity of 0.9 m/s and θ = 0. (**b**) Detail of the pressure along the centerline of the central channel (indicated by an arrow in (**a**)) as a function of the distance from the cylinder’s outer surface for a wind velocity of 0.9 m/s. (**c**) Pressure distribution around the cylinder and inside the channels for wind velocity of 5.3 m/s and θ = 0. (**d**) Detail of the pressure along the centerline of the central channel for a wind velocity of 5.3 m/s.

Preliminary investigation performed by means of 2D FEA (finite element analysis) has been performed on the H3L head in order to check how the hypotheses of (i) uniform pressure gradient along the channels and (ii) uniform pressure distribution inside H_1_ and H_2_ cavities are satisfied. Simulations have been executed using the COMSOL Multiphysics platform with the SST (shear stress transport) turbulence model. Figure 15a shows the pressure distribution for a velocity of 0.9 m/s and a wind direction θ = 0. The pressure inside the central channel (indicated by an arrow in Figure 15a) is shown in Figure 15b as a function of the distance from the channel endpoint at the cylinder’s outer surface. The same plots for a velocity of 5.3 m/s are shown in Figure 15c,d, respectively. These simulations suggest that the pressure gradient inside the channel is not constant and that the pressure in the cavities is not uniform. These non-idealities are much more important at higher wind velocities. In particular, Figure 15d prove that most of the pressure drop occurs in the first quarter of the channel. The results shown in Figure 15a,d should be regarded as a qualitative demonstration that the pressure irregularity inside the channel structure can be the cause of the observed discrepancy with respect of the performances predicted in [32] and, in particular, of the unsatisfactory approximation of the sinusoidal dependence visible in Figure 6b. Quantitative predictions would require a full 3D model.

The resolution of the differential pressure sensors determines the lowest applicable wind velocity: Equation (9) indicates that the peak pressure difference (equal to *H*) should be much larger than the equivalent noise pressure of the sensors to maintain an acceptable angular resolution. A velocity of 0.3 m/s can be considered as a lower bound for the operating range of devices described in this paper. Operation at even smaller velocities can be achieved only using pressure sensors with a lower equivalent noise.

The effect of the cylinder inclination appears only as a uniform attenuation of the pressure differences. A proportionality to the cosine of the inclination angle was found, as in [8]. The capability of detecting the wind direction is not impaired, at least up to a cylinder inclination of 30 degrees, which was the maximum allowed by the set-up. 

Finally, it is important to point out that the approach presented in this work is highly scalable, opening the way to the fabrication of directional wind sensors with even smaller dimensions. Uniform scaling of the heads would result in a proportional variation in the Reynolds number, tied to the cylinder diameter. This should not be a problem, since the detection principle was demonstrated to be applicable over a wide range of Reynolds numbers. The magnitude of the differential pressures does not change significantly with the cylinder diameter, being always of the order of the dynamic pressure, equal to ρ(u_w_)^2^/2. The only critical issue lies in the reduction in the channel diameter, which would increase the pressure loss when, as in the case of this paper, the pressure sensors are actually flow sensors. Sensors characterized by smaller leakage for the same differential pressure can be selected to mitigate this possible cause of signal loss. The selection of smaller pressure sensors can be also adopted to further reduce the overall dimensions of the device.

## Figures and Tables

**Table 1:** Angles and weights used in the devices proposed in this paper and in previous works.

Type	N	Work	Angles (ϕ_k_)	Weights (w_k_)
UDS	1	This work	0, ±45	cos(ϕ_k_)
UW	3	This work	0, ±17°, ±32.5°, ±58.3°	1/7
UDS	3	[29,30]	0, ±22.5, ±45, ±67.5	cos(ϕ_k_)
UW	1	[25]	0, ±21°, ±53°	1/3
UW	2	[28]	0, ±40°	1/5

**Table 2:** Dimensions used for the three heads tested in this work.

Head	Type	*N*	Figure	*L_A0_* (mm)	*L_A1_* (mm)	L_B_ (mm)	*D*_cy_ (mm)	*D*_O_ (mm)	*D*_ch_ (mm)	*h_c_* (mm)
**H3L**	UDS	1	2a	4	5.65	-	20	1	1	3
**H7L**	UW	3	2b	-	-	4	20	1	1	3
**H7S**	UW	3	2b	-	-	2	20	1	1	3

**Table 3:** Summary of performances compared with previous work on anemometers based on the same principle. Errors are measure across the 0.9–8 m/s velocity range.

Type	N	Work	Max Angular Error (deg)	Max Magnitude Deviation
UDS	1	This work, (H3L)	11	20%
UW	3	This work (H7L)	10	13%
UDS	3	[29,30]	12	30%
UW	1	[25]	7 ^1^	8% ^1^
UW	2	[28]	5	8%

^1^ Performances were extrapolated from measurements performed on a single section.

## References

[1] Zhang N., Wang M., Wang N. (2002). Precision agriculture-a worldwide overview. Comput. Electron. Agric..

[2] Joseph G.M.D., Mohammadi M., Sterling M., Baker C.J., Gillmeier S.G., Soper D., Jesson M., Blackburn G.A., Whyatt J.D., Gullick D. (2020). Determination of crop dynamic and aerodynamic parameters for lodging prediction. J. Wind Eng. Ind. Aerodyn..

[3] Bhalchandra B., Shinde G. (2016). Wind Speed and Rain Fall Monitoring System for Precision Agriculture using Low Power Consumption Wireless Sensor Network. Int. J. Agric. Innov. Res..

[4] Catania P., Vallone M. (2020). Application of A Precision Apiculture System to Monitor Honey Daily Production. Sensors.

[5] Li J.G., Cao M.L., Meng Q.H. (2019). Chemical source searching by controlling a wheeled mobile robot to follow an online planned route in outdoor field environments. Sensors.

[6] Burgués J., Hernández V., Lilienthal A.J., Marco S. (2020). Gas distribution mapping and source localization using a 3D grid of metal oxide semiconductor sensors. Sens. Actuators B.

[7] Chen X.X., Huang J. (2019). Odor source localization algorithms on mobile robots: A review and future outlook. Robot. Auton. Syst..

[8] Pan Y., Zhao Z., Zhao R., Fang Z., Wu H., Niu X., Du L. (2019). High accuracy and miniature 2-D wind sensor for boundary layer meteorological observation. Sensors.

[9] Adkins K.A., Swinford C.J., Wambolt P.D., Bease G. (2020). Development of a sensor suite for atmospheric boundary layer measurement with a small multirotor unmanned aerial system. Int. J. Aviat. Aeronaut. Aerosp..

[10] Xing Z.W., Zhang Y.M., Su C.Y., Qu Y.H. Measuring the Horizontal Wind for Forest Fire Monitoring Using Multiple UAVs. Proceedings of the 2019 Chinese Control Conference (CCC).

[11] Hamadi H., Lussier B., Fantoni I., Francis C., Shraim H. Observer-based Super Twisting Controller Robust to Wind Perturbation for Multirotor UAV. Proceedings of the 2019 International Conference on Unmanned Aircraft Systems (ICUAS).

[12] Wang J.Y., Luo B., Zeng M., Meng Q.H. (2018). A wind estimation method with an unmanned rotorcraft for environmental monitoring tasks. Sensors.

[13] Tomić T., Haddadin S. (2020). Towards Interaction, Disturbance and Fault Aware Flying Robot Swarms. Robotics Research.

[14] Arens E., Ghahramani A., Przybyla R., Anderson M., Min S., Peffer T., Raftery P., Zhu M., Luu V., Zhang H. (2020). Measuring 3D indoor air velocity via an inexpensive low-power ultrasonic anemometer. Energy Build..

[15] FT Technologies. https://fttechnologies.com/.

[16] Kim S., Nam T., Park S. (2004). Measurement of flow direction and velocity using a micromachined flow sensor. Sens. Actuators A.

[17] Liu H.B., Lin N., Pan S.S., Miao J., Norford L.K. (2013). High sensitivity, miniature, full 2-D anemometer based on MEMS hot-film sensors. IEEE Sens J..

[18] Zhu Y., Qin M., Ye Y., Yi Z., Long K., Huang Q.A. (2017). Modelling and characterization of a robust, low-power and wide-range thermal wind sensor. Microsyst. Technol..

[19] Shin K.S., Lee D.S., Song S.W., Jung J.P. (2017). Application of surface protective coating to enhance environment-withstanding property of the MEMS 2D wind direction and wind speed sensor. Sensors.

[20] Luo Z., Li Z., Gao C., Hao Y., Jin Y. Anemometer for detection of very low speed air flow with three-dimensional directionality. Proceedings of the 2017 IEEE 12th International Conference on Nano/Micro Engineered and Molecular Systems (NEMS).

[21] Idjeri B., Laghrouche M., Boussey J. (2017). Wind measurement based on MEMS micro-anemometer with high accuracy using ANN technique. IEEE Sens. J..

[22] Barmpakos D., Famelis I.T., Moschos A., Marinatos D., Kaltsas G. (2019). Design and Evaluation of a Multidirectional Thermal Flow Sensor on Flexible Substrate. J. Sens..

[23] Li G.Z., Zhao S., Zhu R. (2019). Wearable anemometer with multi-sensing of wind absolute orientation, wind speed, attitude, and heading. IEEE Sens. J..

[24] Wang S., Yi Z., Qin M., Huang Q.A. (2019). Modeling, simulation, and fabrication of a 2-D anemometer based on a temperature-balanced mode. IEEE Sens. J..

[25] Bruschi P., Dei M., Piotto M. (2009). A low-power 2-D wind sensor based on integrated flow meters. IEEE Sens. J..

[26] Eckman R.M., Dobosy R.J., Auble D.L., Strong T.W., Crawford T.L. (2007). A pressure-sphere anemometer for measuring turbulence and fluxes in hurricanes. J. Atmos. Ocean. Technol..

[27] Sun B., Zhou W., Yuan M.Z., Fang E.Q., Gan N. (2018). A Cylindrical Vehicle-Mounted Anemometer Based on 12 Pressure Sensors—Principle, Prototype Design, and Validation. IEEE Sens J..

[28] Piotto M., Pennelli G., Bruschi P. (2011). Fabrication and characterization of a directional anemometer based on a single chip MEMS flow sensor. Microelectron. Eng..

[29] Bruschi P., Piotto M., Dell’Agnello F., Ware J., Roy N. (2017). Wind speed and direction detection by means of solid-state anemometers embedded on small quadcopters. Procedia Eng..

[30] Bruschi P., Ria A., Piotto M. (2018). A scalable 2D, low power airflow probe for unmanned vehicle and WSN applications. Proceedings of the International Conference on Applications in Electronics Pervading Industry, Environment and Society.

[31] Bruschi P., Nurra V., Piotto M. (2008). A compact package for integrated silicon thermal gas flow meters. Microsyst. Technol..

[32] Bruschi P., Piotto M. (2017). Determination of the wind speed and direction by means of fluidic-domain signal processing. IEEE Sens. J..

[33] Liu C., Du L., Zhao Z. (2016). A directional cylindrical anemometer with four sets of differential pressure sensors. Rev. Sci. Instrum..

[34] Leoni A., Stornelli V., Pantoli L. (2018). A low-cost portable spherical directional anemometer for fixed points measurement. Sens. Actuators A.

[35] Monteiro J., Carrilho J., Silva M.G.D., Miranda A., Silva A. (2017). 3D Printed Pressure Anemometers. 3D Print. Addit. Manuf..

[36] Prudden S., Fisher A., Mohamed A., Watkins S. An anemometer for UAS-based atmospheric wind measurements. Proceedings of the 17th Australian International Aerospace Congress: AIAC 2017.

[37] Al-Rubaiai M., Tsuruta R., Gandhi U., Wang C., Tan X. (2019). A 3D-printed stretchable strain sensor for wind sensing. Smart Mater. Struct..

[38] Shin H.S., Kim T., Bergbreiter S., Park Y.L. Biomimetic Soft Airflow Sensor with Printed Ionogel Conductor. Proceedings of the 2019 2nd IEEE International Conference on Soft Robotics (RoboSoft).

[39] Daniel F., Peyrefitte J., Radadia A.D. (2020). Towards a Completely 3D Printed Hot Wire Anemometer. Sens. Actuators A.

[40] Barile G., Leoni A., Fern G. A Differential Capacitive Multi-Material 3D Printed Sensor for Portable Anemometric Applications. Proceedings of the 2019 IEEE 8th International Workshop on Advances in Sensors and Interfaces (IWASI).

[41] Formlabs. https://formlabs.com/it/.

[42] FreeCAD. https://www.freecadweb.org/.

[43] Sensirion Series SDP800 Differential Pressure Sensors, Sensirion Catalogue. https://www.sensirion.com.

